# Report upon the Hemostatic Virtues of the Brocchieri Water and Ergotine

**Published:** 1846-12

**Authors:** 


*Report upon the Hemostatic Virtues of the Brocchieri Water and Ergotine.*—The editors of the *Southern Journal of Medicine and Pharmacy* have made an interesting report on this subject, in which they come to the following conclusions:

From the numerous experiments performed, and from the manner in which they were modified, as well as the care bestowed upon them, without reference to their favorable or unfavorable results, we are warranted in coming to the following conclusions:

1st. That when Brocchieri Water, ergotine, or a watery emulsion of creosote, is applied to the divided artery of a sheep, it depends greatly, if not altogether, upon the manner in which the lint is applied to the wound on the artery, whether the hemorrhage is arrested or not. If it be placed immediately upon the orifice of the cut vessel, the success is certain; if, however, the vessel shrink from contact with the lint, the animal is almost certain to bleed to death. The former is shown by the experiments 4 and 5 of the first report, and by 3, 5 and 6 in the present one; and the latter, by experiments 1, 2, and 4, in this report.

2d. That by a small pledget of *simple lint*, placed immediately upon the incision made into a sheep's carotid artery, that the homorrhage is arrested in a few moments; and after a lapse of from twenty to thirty minutes, the animal may be let loose, without any apprehension of the return of the hemorrhage. If the lint be applied so as not to touch the wound in the artery, all effort to arrest the hemorrhage becomes ineffectual; this is proved by reference to experiments 1, 2 and 3 of the first report, while experiments 7, 8 and 9 give evidence of what was first stated. From these results, it will be seen how many difficulties often attend the simplest experiments, and how important it is to leave no point, not the most apparently trivial, without close examination; it is true, it requires time and trouble, but both are more than compensated for by a knowledge that we become in possession of truths that are important to ourselvs and to others.

3d. The sheep is an unfit animal to try the hemostatic power of substances, with reference to what their fitness may be in the case of the human subject; for although sheep will bleed to death by a wound in one of the larger arteries, still, by the application of a small pledget of lint, sustained with a little pressure *immediately upon the wound in the vessel*, the hemorrhage will cease, and the animal survive. The same we are convinced may be said of all like experiments upon the lower class of animals, as in many of them the hemorrhage from the larger vessel will be arrested spontaneously; this is true of the dog; in fact, so far as our knowledge extends, the sheep is more readily bled to death than almost any other of the quadrupeds. Furthermore, the blood of animals is more plastic, coagulating with far greater rapidity than that of man; and as the arresting of the hemorrhage in these experiments is dependent upon the formation of a clot around the opening and in the cavity of the vessel, it ought therefore to happen more readily in them than in man.

4th. If the hemostatic virtues of the agents already alluded to, are to be correctly ascertained, it is only *by experiments upon the human subject*, and no value should be given to those made in any other way. Whether the Brocchieri water, ergotine and creosote will stand this test, we are not as yet prepared to say, although several experiments made with them have come to our notice, but they are of so contradictory a character, that no definite conclusion can be formed. These substances no doubt hasten the coagulation of blood, and may, under some circumstances, arrest hemorrhage coming from the smaller arteries; but in the case of the larger vessels, they are of no manner of use, at least not more than the lint without them.

The experiments on the human subject that have come to our notice are,—wound on the hand, oozing, some time after the operation of hydrocele; oozing from a tumour on the back;—tried with Brocchieri water. In the first case, there appeared to be no effect; in the last two, some slight effect; the oozing in the case of the hydrocele, although diminished, could not be arrested. In a case of hemorrhoids that was operated upon, application of the ergotine was made with apparently good effect. In two cases of operation for hydrocele, the emulsion of creosote was used; in one of them, the bleeding from the small vessels was arrested; in the other no effect. So it is clear, that as yet, there is no danger of ligatures being supplanted by either of the above agents.

